# Association between the cardiometabolic index and NAFLD and fibrosis

**DOI:** 10.1038/s41598-024-64034-3

**Published:** 2024-06-08

**Authors:** Laisha Yan, Xiaoyan Hu, Shanshan Wu, Can Cui, Shunying Zhao

**Affiliations:** Department of Cardio Surgery Intensive Care Unit, Ningbo Medical Centre Li Huili Hospital, Ningbo, China

**Keywords:** NAFLD, Hepatic steatosis, Liver fibrosis, NHANES, Cardiometabolic index, Endocrinology, Medical research

## Abstract

Composed of obesity and lipid parameters, the cardiometabolic index (CMI) has emerged as a novel diagnostic tool. Originally developed for diabetes diagnosis, its application has expanded to identifying patients with cardiovascular diseases, such as atherosclerosis and hypertension. However, the relationship between CMI and non-alcoholic fatty liver disease (NAFLD) and liver fibrosis in the US population remains unclear. This cross-sectional study analyzed data from the National Health and Nutrition Examination Survey (NHANES) spanning 2017–2020, involving 2996 participants aged 20 years or older. Vibration controlled transient elastography using a FibroScan® system (model 502, V2 Touch) with controlled attenuation parameter measurements identified NAFLD at a threshold of ≥ 274 dB/m, while liver stiffness measurement (LSM) results (median, ≥ 8.2 kPa) indicated fibrosis. A multifactorial logistic regression model explored the relationship between CMI and NAFLD and fibrosis. The effectiveness of CMI in detecting NAFLD and liver fibrosis was assessed through receiver operating characteristic curve analysis. Controlling for potential confounders, CMI showed a significant positive association with NAFLD (adjusted OR = 1.44, 95% CI 1.44–1.45) and liver fibrosis (adjusted OR = 1.84, 95% CI 1.84–1.85). The Areas Under the Curve for predicting NAFLD and fibrosis were 0.762 (95% CI 0.745 ~ 0.779) and 0.664(95% CI 0.633 ~ 0.696), respectively, with optimal cut-off values of 0.462 and 0.527. There is a positive correlation between CMI and NAFLD and fibrosis, which is a suitable and simple predictor of NAFLD and fibrosis.

## Introduction

Nonalcoholic fatty liver disease (NAFLD) is a primary cause of cirrhosis and hepatocellular carcinoma and has significant socioeconomic impacts^1^. NAFLD predominantly manifests as hepatic steatosis, lobular inflammation, hepatocyte ballooning, and fibrosis^2^. The recently published multisociety Delphi consensus statement on the new fatty liver disease nomenclature, which replaces NAFLD with metabolic dysfunction-associated steatotic liver disease (MASLD), clearly reveals the etiology of the disease and the risk factors^3^. It is considered the liver’s expression of metabolic syndrome^1,4,5^.

Even though liver biopsy stands as the gold standard for NAFLD diagnosis, its invasive characteristics frequently limit its acceptance. As a result, transient elastography, a non-invasive technique, has gained substantial attention due to its clinical utility^6^. This technique concurrently assesses both the median liver stiffness measurement (LSM) and controlled attenuation parameter (CAP), shedding light on hepatic steatosis and fibrosis^6^.

First introduced in 2015, the cardiometabolic index (CMI) is derived from the product of the waist-to-height ratio (WHtR) and the triglyceride (TG) to high-density lipoprotein cholesterol (HDL-C) ratio. It has been proposed as a predictive tool for assessing risks associated with type 2 diabetes and cardiovascular diseases^7^. Studies have identified correlations between the CMI and conditions such as hyperuricaemia, obstructive sleep apnoea, and stroke^8–10^. Given the promising diagnostic capability of the CMI for various metabolic disorders, its potential role in NAFLD diagnosis warrants further investigation. This hypothesis has been verified among Asian populations, yet comprehensive research remains limited in US populations^11^. Moreover, no studies have yet explored the correlation between the CMI and liver fibrosis.

In this study, data from the National Health and Nutrition Examination Survey (NHANES) was utilized to examine the correlation between the CMI and NAFLD and fibrosis.

## Methods

### Study population

**Figure 1:**
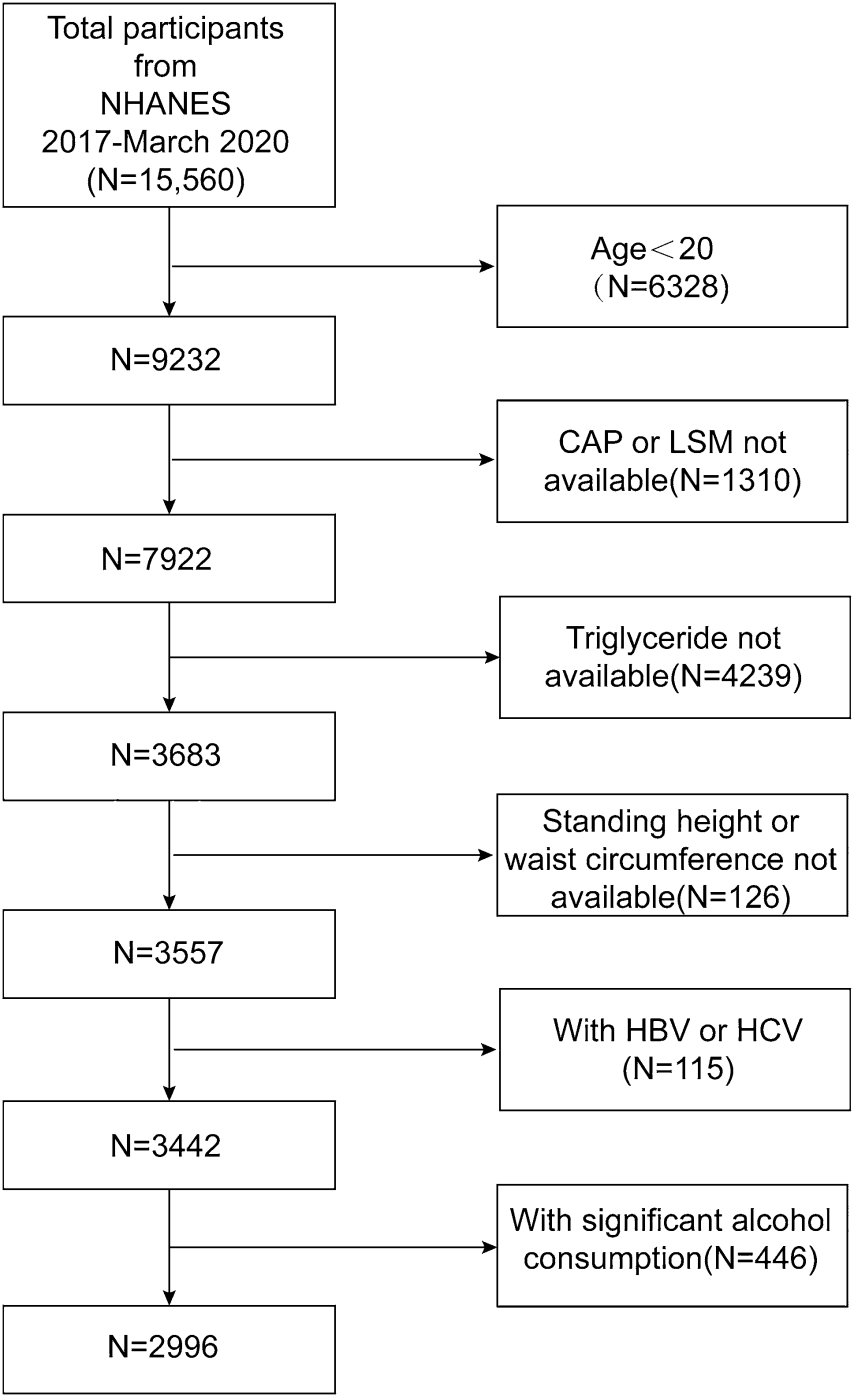
Flowchart of participant selection.

The NHANES serves as a nationally representative database, capturing the health and nutritional status of U.S. children and adults. Analysis of NHANES datasets from 2017 to 2020 revealed data for 15,560 participants. The analytical sample was narrowed down to 2996 subjects after applying the following exclusion criteria: individuals below the age of 20 years, individuals with a history of heavy alcohol consumption (defined as the daily intake of 4 or 5 or more drinks), subjects diagnosed with hepatitis B or C, and participants with incomplete laboratory or examination data. The inclusion and exclusion criteria are depicted in Fig. 1.

### Data collection

The outcome variables of the study were NAFLD and liver fibrosis, with the CMI value serving as the independent variable. The CMI value was determined using the following formula: TG/HDL-C × WHtR. Utilizing a FibroScan Model 502 V2 Touch system, trained NHANES staff acquired participants’ LSM and CAP values through vibration-controlled transient elastography (VCTE) assessments. A CAP value ≥ 274 dB/m was indicative of hepatic steatosis, whereas an LSM ≥ 8.2 kPa was indicative of fibrosis^12^.

Additional variables were collected based on previous research findings and clinical expertise^13,14^. They encompassed the following demographic, health, and biochemical markers: Age, sex, race, body mass index (BMI), smoking status, diabetes, hypertension, total cholesterol (TC), low-density lipoprotein cholesterol (LDL-C), alanine aminotransferase (ALT), albumin (ALB), gamma-glutamyl transferase (GGT), aspartate aminotransferase (AST), blood urea nitrogen (BUN), creatinine (Cr), Total bilirubin (TB), uric acid (UA).

### Statistical analysis

To account for the complex sampling design of the NHANES, we incorporated weights (WTSAFPRP) into our analysis, in accordance with recommendations from the NHANES official website. Data management and statistical analyses were performed using SAS 9.4(version 9.4 for Windows, SAS Institute, Inc., Cary, NC, USA). Qualitative data were described using n (%), while quantitative data that did not follow a normal distribution were described using Median (P25, P75). Comparisons between groups were conducted using the rank-sum test. A binary logistic regression analysis was utilized to examine the relationship between CMI and NAFLD and fibrosis. In the multivariate analysis, considering the intercorrelation among variables, the Variance Inflation Factor (VIF) was employed to assess multicollinearity. To control for potential confounding factors that might affect the results, a series of models were established: Model 1, which did not adjust for any confounding factors; Model 2, which adjusted for gender, age, BMI, and race based on Model 1; Model 3, which further adjusted for smoking, diabetes, and hypertension based on Model 2; Model 4, which incorporates adjustments for all non-collinear variables including TC, AST, GGT, ALB, BUN, TB, and UA, building upon the adjustments made in Model 3. A *P*-value of ≤ 0.05 was considered statistically significant. The diagnostic efficacy of CMI for detecting NAFLD and fibrosis was assessed through receiver operating characteristic (ROC) curve analysis.

### Ethical statement

The research involving human participants underwent a thorough review and received approval from the Research Ethics Review Board of the NCHS. All patients or participants gave their written informed consent to be part of this study.

### Subject characteristics

A total of 2996 adults, identified via the established inclusion and exclusion criteria, constituted the study cohort. The cohort’s demographics and key clinical characteristics are as follows. The mean age was 49.98 ± 17.33 years. Of the participants, 45.23% were males and 54.77% were females. A total of 13.05% of the participants were Mexican American, 10.35% were Other Hispanic, 33.14% were Non-Hispanic White, 24.67% were Non-Hispanic Black, 18.79% were from other racial groups. NAFLD was diagnosed in 1311 individuals and liver fibrosis in 275, constituting 43.76% and 9.18% of the study population, respectively.

Compared to non-NAFLD individuals, those with NAFLD were more likely to be older, male, and of Mexican American ethnicity. They also exhibited higher rates of smoking, diabetes, and hypertension, along with significantly elevated levels of BMI, TG, TC, LDL-C, GGT, ALT, AST, BUN, UA, Cr, and TB. In contrast, their ALB and HDL-C levels were significantly lower. CMI values, on average, were significantly lower in individuals without NAFLD compared to those with the condition (0.32 vs 0.71, *P* < 0.001). Additionally, a greater proportion of NAFLD patients were found in the highest CMI quartiles (Q3 and Q4), a significant difference compared to the non-NAFLD group (*P* < 0.001). These findings are detailed in Table 1.

**Table 1:** Clinical and biochemical characteristics of the study subjects with or without NAFLD.

		Non-NAFLD	NAFLD	
	Total	CAP < 274 dB/m (n = 1685)	CAP ≥ 274 dB/m (n = 1311)	*P*
CMI [M (P_25_, P_75_)]	0.46 (0.25,0.81)	0.32 (0.20,0.54)	0.71 (0.43,1.11)	< 0.001#
CMI quartile				
Q1 (≤ 0.25)	749 (25.00)	634 (37.63)	115 (8.77)	< 0.001
Q2 (0.25 ~ 0.46)	749 (25.00)	504 (29.91)	245 (18.69)	
Q3 (0.46 ~ 0.81)	749 (25.00)	340 (20.18)	409 (31.20)	
Q4 (> 0.81)	749 (25.00)	207 (12.28)	542 (41.34)	
Sex				
Men	1355 (45.23)	711 (42.20)	644 (49.12)	< 0.001
Women	1641 (54.77)	974 (57.80)	667 (50.88)	
Age [M (P_25_, P_75_)]	51.0 (35.0,64.0)	46.0 (32.0,62.0)	54.0 (41.0,65.0)	< 0.001#
BMI [M (P_25_, P_75_)]	28.6 (24.7,33.5)	26.0 (23.0,29.9)	32.0 (28.2,37.1)	< 0.001#
Race				
Mexican American	391 (13.05)	166 (9.85)	225 (17.16)	< 0.001
Other Hispanic	310 (10.35)	173 (10.27)	137 (10.45)	
Non-Hispanic white	993 (33.14)	534 (31.69)	459 (35.01)	
Non-Hispanic black	739 (24.67)	474 (28.13)	265 (20.21)	
Other race—Including multi-racial	563 (18.79)	338 (20.06)	225 (17.16)	
Smoke				
Yes	1121 (37.44)	599 (35.59)	522 (39.82)	< 0.001
No	1873 (62.56)	1084 (64.41)	789 (60.18)	
Diabetes				
Yes	448 (14.96)	150 (8.91)	298 (22.73)	< 0.001
No	2546 (85.04)	1533 (91.09)	1013 (77.27)	
Hypertension				
Yes	1099 (36.74)	475 (28.26)	624 (47.63)	< 0.001
No	1892 (63.26)	1206 (71.74)	686 (52.37)	
TG (mmol/L) [M (P_25_, P_75_)]	1.01 (0.68,1.50)	0.81 (0.60,1.21)	1.30 (0.90,1.84)	< 0.001#
TC (mmol/L) [M (P_25_, P_75_)]	4.68 (4.06,5.40)	4.63 (4.01,5.33)	4.76 (4.14,5.51)	< 0.001#
HDL-C (mmol/L) [M (P_25_, P_75_)]	1.32 (1.09,1.60)	1.42 (1.19,1.71)	1.19 (1.03,1.45)	< 0.001#
LDL-C (mmol/L) [M (P_25_, P_75_)]	2.77 (2.20,3.36)	2.69 (2.17,3.31)	2.85 (2.25,3.44)	< 0.001#
AST(IU/L) [M (P_25_, P_75_)]	19.0 (15.0,23.0)	18.0 (15.0,22.0)	19.0 (16.0,25.0)	< 0.001#
ALT(IU/L) [M (P_25_, P_75_)]	17.0 (13.0,25.0)	15.0 (11.0,21.0)	21.0 (15.0,30.0)	< 0.001#
GGT(IU/L) [M (P_25_, P_75_)]	21.0 (15.0,30.0)	18.0 (13.0,25.0)	24.0 (18.0,37.0)	< 0.001#
ALB(g/L) [M (P_25_, P_75_)]	40.0 (38.0,42.0)	41.0 (39.0,43.0)	40.0 (38.0,42.0)	< 0.001#
BUN (mmol/L) [M (P_25_, P_75_)]	5.0 (3.9,6.1)	4.6 (3.9,6.1)	5.0 (3.9,6.1)	< 0.001#
Cr(mmol/L) [M (P_25_, P_75_)]	73.4 (61.9,86.6)	73.4 (61.9,87.5)	74.4 (61.0,88.8)	< 0.001#
TB (umol/L) [M (P_25_, P_75_)]	6.8 (5.1,10.3)	6.8 (5.1,10.3)	7.1 (5.1,10.4)	< 0.001#
UA (umol/L) [M (P_25_, P_75_)]	315.2 (261.7, 374.7)	297.4 (249.8, 350.9)	339.0 (285.5, 398.5)	< 0.001#

Values are n (%) or mean (standard deviation) or median (P25, P75).

NAFLD, nonalcoholic fatty liver disease; TC, total cholesterol; HDL-C, high-density lipoprotein cholesterol; TG, triglyceride; LDL-C, low-density lipoprotein cholesterol; ALT, alanine aminotransferase; ALB, albumin; GGT, gamma-glutamyl transferase; AST, aspartate aminotransferase; BUN, blood urea nitrogen; Cr, creatinine; TB, total bilirubin; UA, uric acid; LSM, liver stiffness measurement; CMI, cardiometabolic index.

"#" indicates that the rank-sum test was used.

Cirrhosis patients, more likely to be older, Mexican American, and smokers, exhibited higher rates of diabetes and hypertension, along with elevated levels of BMI, TG, ALT, AST, GGT, BUN, Cr, TB, and UA. Conversely, their LDL-C, HDL-C, ALB, and TC levels were lower. The CMI was significantly higher in the hepatic fibrosis group than in individuals without hepatic fibrosis (0.73 vs 0.44, *P* < 0.001), with a larger proportion found in the highest CMI quartiles (Q3, Q4, *P* < 0.001), as detailed in Table 2.

**Table 2:** Clinical and biochemical characteristics of the study subjects with or without liver fibrosis.

		Normal	Liver fibrosis	
	Total	< 8.2kPa (n = 2721)	≥ 8.2kPa (n = 275)	*P*
CMI [M (P_25_, P_75_)]	0.46 (0.25,0.81)	0.44 (0.24,0.77)	0.73 (0.43,1.11)	< 0.001#
CMI quartile				
Q1 (≤ 0.25)	749 (25.00)	724 (26.61)	25 (9.09)	< 0.001
Q2 (0.25 ~ 0.46)	749 (25.00)	698 (25.65)	51 (18.55)	
Q3 (0.46 ~ 0.81)	749 (25.00)	676 (24.84)	73 (26.55)	
Q4 (> 0.81)	749 (25.00)	623 (22.90)	126 (45.82)	
Sex				
Men	1355 (45.23)	1209 (44.43)	146 (53.09)	< 0.001
Women	1641 (54.77)	1512 (55.57)	129 (46.91)	
Age [M (P_25_, P_75_)]	51.0 (35.0,64.0)	50.0 (34.0,63.0)	58.0 (43.0,67.0)	< 0.001#
BMI [M (P_25_, P_75_)]	28.6 (24.7,33.5)	28.1 (24.5,32.6)	35.8 (29.3,42.0)	< 0.001#
Race				
Mexican American	391 (13.05)	346 (12.72)	45 (16.36)	< 0.001
Other Hispanic	310 (10.35)	283 (10.40)	27 (9.82)	
Non-Hispanic white	993 (33.14)	892 (32.78)	101 (36.73)	
Non-Hispanic black	739 (24.67)	676 (24.84)	63 (22.91)	
Other race—Including multi-racial	563 (18.79)	524 (19.26)	39 (14.18)	
Smoke				
Yes	1121 (37.44)	1004 (36.93)	117 (42.55)	< 0.001
No	1873 (62.56)	1715 (63.07)	158 (57.45)	
Diabetes				
Yes	448 (14.96)	337 (12.39)	111 (40.36)	< 0.001
No	2546 (85.04)	2382 (87.61)	164 (59.64)	
Hypertension				
Yes	1099 (36.74)	941 (34.65)	158 (57.45)	< 0.001
No	1892 (63.26)	1775 (65.35)	117 (42.55)	
TG (mmol/L) [M (P_25_, P_75_)]	1.01 (0.68,1.50)	0.98 (0.67,1.49)	1.22 (0.85,1.75)	< 0.001#
TC (mmol/L) [M (P_25_, P_75_)]	4.68 (4.06,5.40)	4.71 (4.11,5.43)	4.37 (3.78,5.15)	< 0.001#
HDL (mmol/L) [M (P_25_, P_75_)]	1.32 (1.09,1.60)	1.34 (1.11,1.63)	1.16 (1.03,1.40)	< 0.001#
LDL (mmol/L) [M (P_25_, P_75_)]	2.77 (2.20,3.36)	2.77 (2.22,3.39)	2.48 (1.94,3.23)	< 0.001#
AST(IU/L) [M (P_25_, P_75_)]	19.0 (15.0,23.0)	19.0 (15.0,23.0)	21.0 (17.0,29.0)	< 0.001#
ALT (IU/L) [M (P_25_, P_75_)]	17.0 (13.0,25.0)	17.0 (12.0,24.0)	23.0 (15.0,35.0)	< 0.001#
GGT(IU/L) [M (P_25_, P_75_)]	21.0 (15.0,30.0)	20.0 (14.0,29.0)	30.0 (20.0,54.0)	< 0.001#
ALB(g/L) [M (P_25_, P_75_)]	40.0 (38.0,42.0)	40.0 (38.0,42.0)	39.0 (37.0,42.0)	< 0.001#
BUN (mmol/L) [M (P_25_, P_75_)]	5.0 (3.9,6.1)	5.0 (3.9,6.1)	5.4 (4.3,6.8)	< 0.001#
Cr(mmol/L) [M (P_25_, P_75_)]	73.4 (61.9,86.6)	73.4 (61.9,86.6)	75.1 (62.8,90.2)	< 0.001#
TB (umol/L) [M (P_25_, P_75_)]	6.8 (5.1,10.3)	6.8 (5.1,10.3)	8.6 (5.1,10.3)	< 0.001#
UA (umol/L) (®x ± s)	321.9 ± 85.3	318.6 ± 84.6	354.2 ± 84.6	< 0.001

Values are n (%) or mean (standard deviation) or median (P25, P75).

"#" indicates that the rank-sum test was used.

### Association between CMI and NAFLD

In the multivariate logistic regression model, CMI was significantly positively associated with NAFLD, and this association remained consistent across various models: the unadjusted model (Model 1), the minimally adjusted model (Model 2), the partially adjusted model (Model 3), and the fully adjusted model for all non-collinear variables (Model 4). In the fully adjusted model, an increase of one unit in CMI was associated with a 44% increase in the risk of NAFLD (adjusted OR: 1.44; 95% CI 1.44, 1.45). Furthermore, compared to the first quartile of CMI, the risk of NAFLD for subjects in the second, third, and fourth quartiles increased by 2.53, 6.92, and 14.48 times, respectively, with these results remaining robust after stepwise adjustment for confounding factors (Table 3).

**Table 3:** Association between CMI and NAFLD.

	Modle1		Modle2		Modle3		Modle4	
	OR (95%CI)	*P*	OR (95%CI)	*P*	OR (95%CI)	*P*	OR (95%CI)	*P*
CMI	8.73 (8.72 ~ 8.73)	< 0.001	3.47 (3.46 ~ 3.47)	< 0.001	3.34 (3.33 ~ 3.34)	< 0.001	1.44 (1.44 ~ 1.45)	< 0.001
CMI quartile								
Q1 (≤ 0.25)	1.00 (ref)		1.00 (ref)		1.00 (ref)		1.00 (ref)	
Q2 (0.25 ~ 0.46)	2.53 (2.53 ~ 2.54)	< 0.001	1.27 (1.27 ~ 1.28)	< 0.001	1.24 (1.24 ~ 1.25)	< 0.001	0.90 (0.90 ~ 0.91)	< 0.001
Q3 (0.46 ~ 0.81)	6.92 (6.92 ~ 6.93)	< 0.001	2.90 (2.90 ~ 2.91)	< 0.001	2.75 (2.74 ~ 2.75)	< 0.001	1.37 (1.37 ~ 1.38)	< 0.001
Q4 (> 0.81)	14.48 (14.46 ~ 14.49)	< 0.001	4.36 (4.36 ~ 4.37)	< 0.001	4.09 (4.09 ~ 4.10)	< 0.001	0.97 (0.97 ~ 0.98)	< 0.001

Model 1 is unadjusted. Model 2 is adjusted for gender, age, race, and BMI. Model 3 is further adjusted for smoking, diabetes, and hypertension based on Model 2. Model 4 is additionally adjusted for TC, AST, GGT, ALB, BUN, TB, and UA based on Model 3.

### Association between CMI and liver fibrosis

The relationship between higher CMI levels and increased liver fibrosis risk was notably strong and positive, maintaining significance even after adjusting for all non-collinear covariates (adjusted OR: 1.84; 95% CI 1.84, 1.85). Analysis by CMI quartiles revealed a progressive increase in liver fibrosis risk with higher quartiles: individuals in Q2, Q3, and Q4 experienced a 2.23, 3.32, and 6.10 times greater risk, respectively, compared to those in Q1. This pattern of association persisted even after comprehensive stepwise adjustment for potential confounders, as elaborated in Table 4.

**Table 4:** Association between CMI and hepatic fibrosis.

	Modle1		Modle2		Modle3		Modle4	
	OR (95%CI)	*P*	OR (95%CI)	*P*	OR (95%CI)	*P*	OR (95%CI)	*P*
CMI	1.37 (1.37 ~ 1.37)	< 0.001	1.08 (1.08 ~ 1.08)	< 0.001	1.05 (1.05 ~ 1.05)	< 0.001	1.84 (1.84 ~ 1.85)	< 0.001
CMI quartile								
Q1 (≤ 0.25)	1.00 (ref)		1.00 (ref)		1.00 (ref)		1.00 (ref)	
Q2 (0.25 ~ 0.46)	2.23 (2.22 ~ 2.23)	< 0.001	1.19 (1.19 ~ 1.20)	< 0.001	1.08 (1.08 ~ 1.09)	< 0.001	1.18 (1.18 ~ 1.19)	< 0.001
Q3 (0.46 ~ 0.81)	3.32 (3.31 ~ 3.33)	< 0.001	1.27 (1.27 ~ 1.28)	< 0.001	1.04 (1.04 ~ 1.05)	< 0.001	1.22 (1.22 ~ 1.23)	< 0.001
Q4 (> 0.81)	6.10 (6.09 ~ 6.11)	< 0.001	1.65 (1.64 ~ 1.65)	< 0.001	1.28 (1.27 ~ 1.28)	< 0.001	1.74 (1.74 ~ 1.75)	< 0.001

Model 1 is unadjusted. Model 2 is adjusted for gender, age, race, and BMI. Model 3 is further adjusted for smoking, diabetes, and hypertension based on Model 2. Model 4 is additionally adjusted for TC, AST, GGT, ALB, BUN, TB, and UA based on Model 3.

### The ability of CMI to detect NAFLD and liver fibrosis

**Figure 2:**
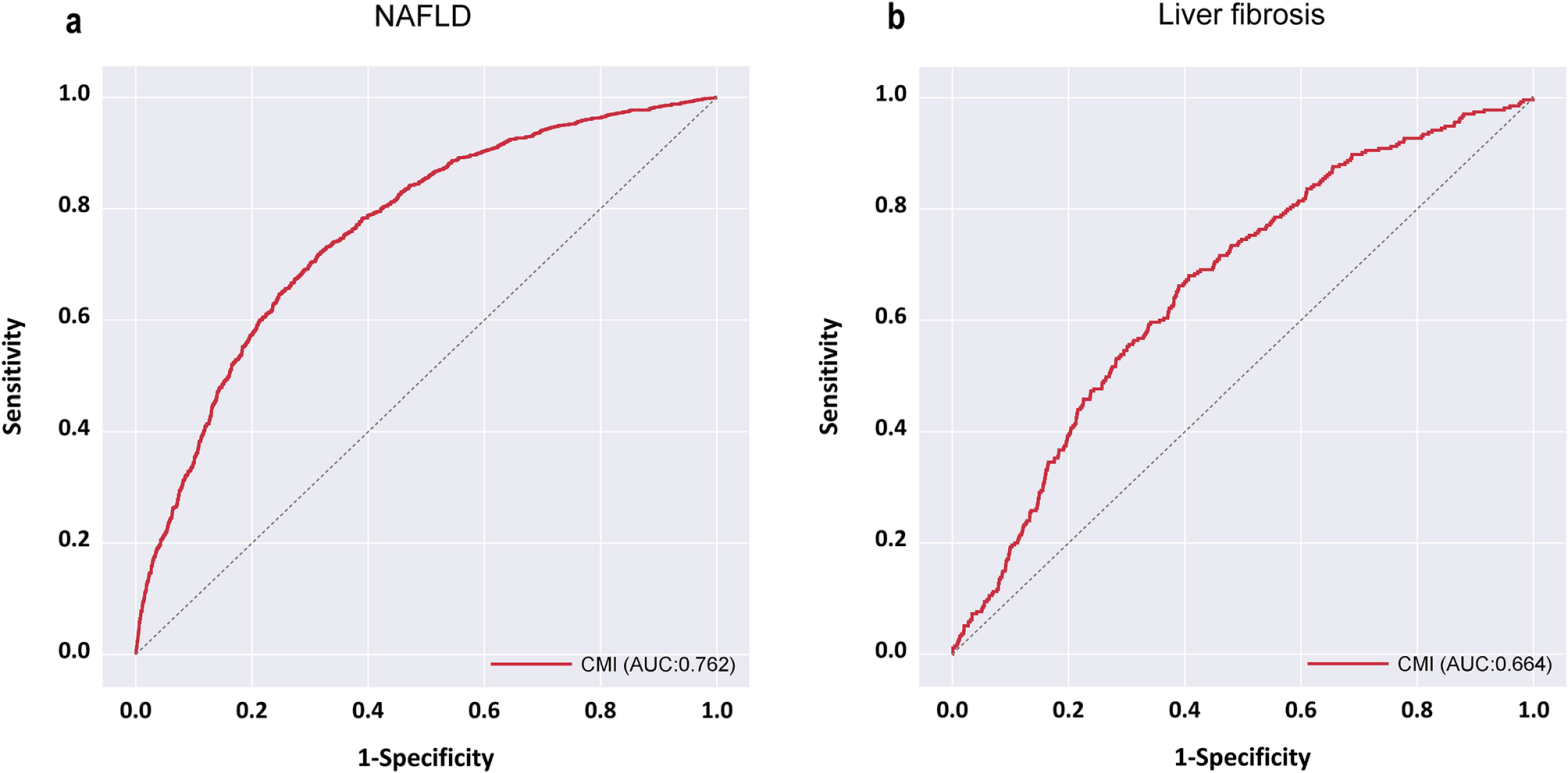
ROC Curves for CMI in Diagnosing NAFLD and liver fibrosis (**a**) NAFLD (**b**) liver fibrosis.

To evaluate the predictive accuracy of the CMI for NAFLD and liver fibrosis, ROC curve analysis was performed. The Areas Under the Curve (AUC) for predicting NAFLD using CMI, depicted in Fig. 2a, was 0.762(95% CI 0.745 ~ 0.779). For liver fibrosis prediction with CMI, shown in Fig. 2b, the AUC was 0.664(95% CI 0.633 ~ 0.696). Detailed analyses, including optimal cutoff values and their corresponding sensitivity and specificity, are presented in Table 5. Specifically, the optimal cutoff for NAFLD prediction was identified as 0.462, yielding a sensitivity of 72.2% and a specificity of 68.1%. For liver fibrosis, the cutoff was established at 0.527, resulting in a sensitivity of 68.0% and a specificity of 59.3%.

**Table 5:** CMI performance metrics for NAFLD and fibrosis screening.

	AUC	*Z*	*P*	Cut Off	Sen	Spe	Youden	PPV	NPV
NAFLD	0.762 (0.745 ~ 0.779)	30.02	< 0.001	0.462	0.722	0.681	0.404	0.638	0.759
Liver fibrosis	0.664 (0.633 ~ 0.696)	10.12	< 0.001	0.527	0.680	0.593	0.273	0.144	0.948

Sen, sensitivity; Spe, specificity; Youden, Youden’s Index; PPV, positive predictive value; NPV, negative predictive value.

## Discussion

To our knowledge, this cross-sectional analysis represents the first extensive clinical investigation into the association between the CMI and both NAFLD and liver fibrosis in the U.S. population, involving 2996 participants. We discovered a significant positive correlation between CMI and both NAFLD and liver fibrosis, persisting even after adjustment for potential confounders through multivariate logistic regression. With AUC of 0.762 for NAFLD and 0.664 for liver fibrosis, our results suggest that CMI serves as an effective predictive marker for these conditions, indicating good diagnostic performance.

Introduced in 2015, the CMI is a novel marker derived from obesity and lipid profiles. Initially used in diabetes diagnosis, the CMI showed a robust correlation with hyperglycaemia and diabetes in both sexes, with notable sex-specific differences^7^. Another prospective study in middle-aged and older Chinese adults showed the same results: A positive association was observed between the CMI and the risk of new-onset type 2 diabetes in middle-aged and older Chinese adults, with a high CMI value recognized as a contributing factor to the development of type 2 diabetes^15^. In an analysis of 174,698 adults, there was a notable correlation between the CMI and hyperuricaemia. This association proved to be more robust than connections with other indices, such as body fat percentage, BMI, the body roundness index, and the visceral fat index^16^. The new MASLD definition emphasizes the significant impact of cardiometabolic risk factors (overweight or obesity, elevated blood glucose, low high-density lipoprotein cholesterol, hypertension, and hypertriglyceridemia) on the development and progression of fatty liver disease. It is inferred that the CMI can also serve as a predictive marker for NAFLD. Recently, a correlation between NAFLD and the CMI was found in a Chinese cohort study. After adjusting for potential confounding factors, a higher CMI value was independently associated with NAFLD. For every standard deviation increase in the CMI value, the risk of non-alcoholic fatty liver disease increases by 28%^11^. In another study of 943 Chinese participants, similar findings were demonstrated. Further subgroup analyses showed significant interactions between the CMI and the risk of MAFLD in terms of sex, age, and BMI^17^. Previous research has investigated the relationship between CMI and the incidence of NAFLD in Asian populations. However, it remains uncertain whether this correlation exists in other ethnic groups. Moreover, while NAFLD is prevalent in the general population, only a limited subset progresses to advanced liver fibrosis. The precise identification of this subset is crucial from a clinical standpoint. Existing literature underlines that fibrosis staging is the primary predictor of both overall and liver-specific mortality in NAFLD patients^18^. Previous indicators used to assess hepatic steatosis, such as the fatty liver index (FLI) and hepatic steatosis index (HSI), along with the NAFLD Fibrosis Score used for measuring fibrosis, are relatively complex to calculate and less suitable for clinical application. There is a need for simpler indicators to screen the target population for further examination.

There is a strong association between obesity and NAFLD progression^19^, with central obesity posing a greater risk than peripheral obesity^20–22^. Visceral fat accumulation plays a partial role in causing hepatic steatosis in overweight and obese individuals, with females being particularly affected^23^. The severity of hepatic steatosis correlates positively with visceral and subcutaneous abdominal adiposity^24^. This relationship is evident not only in hepatic steatosis but also in the progression of hepatic fibrosis. A longitudinal study indicated that abdominal adiposity was the primary risk factor associated with changes in LSM values and the progression of moderate to advanced liver fibrosis in the cohort^25^. Gastric weight-loss surgery has been shown to significantly alleviate hepatic steatosis and fibrosis^26^. Visceral adipose tissue, characterized by heightened lipolysis and insulin resistance, supplies the liver with free fatty acids (FFAs) that are subsequently esterified into TG^27^. Furthermore, this tissue releases pro-inflammatory mediators such as tumor necrosis factor-α (TNF-α) and Interleukin-6 (IL-6), fostering insulin resistance. Such inflammatory mediators initiate macrophage infiltration, activate Kupffer cells, and stimulate hepatic stellate cells, leading to the secretion of extracellular matrix proteins and subsequent fibrotic progression^28,29^. For the assessment of abdominal obesity, the WHtR is recognized as a robust measure. The WHtR is based on waist circumference, and its sensitivity is not affected by height, offering easy computation and consistency across populations. Compared to traditional metrics such as BMI, the WHtR provides a more precise gauge of abdominal obesity^30,31^. Strong associations have been identified between the WHtR and fatty liver manifestations in paediatric and adolescent cohorts^32^.

In epidemiology, hepatic steatosis has been associated with insulin resistance^33,34^. Hepatic steatosis results in insulin resistance, and the converse is also true. Steatotic livers further the worsening of insulin resistance by hindering the removal of insulin from portal blood, thereby maintaining a continuous cycle of deterioration. Insulin resistance also stands out as a pivotal factor in the pathogenesis and natural progression of NAFLD^35^. An imbalance in the production of TNF-α, IL-6, leptin, free fatty acids, and adiponectin leads to insulin resistance and inflammation, which are the primary pathophysiologies for liver fibrosis in patients with fatty liver^36^. Among type 2 diabetes patients, insulin resistance is identified as an independent risk factor associated with liver fibrosis^37^. A study by Ercin CN on 215 biopsy-confirmed NAFLD male patients suggests that insulin resistance values, rather than visceral adiposity index values, are independently correlated with liver fibrosis^38^. The ratio of TG to HDL-C serves as an indicative tool for insulin resistance^39–42^. The association between insulin resistance and TGs as well as the TG/HDL-C ratio is more significant in women than in men^43^. Multiple studies have highlighted the efficacy of the TG/HDL-C ratio in predicting NAFLD^44–46^, a fact further corroborated by Fan et al.’s cross-sectional analysis, which emphasized the significant correlation of the TG/HDL-C ratio with NAFLD risk in healthy subjects^47^.

### Study strengths and limitations

The study has several strengths that enhance the credibility and validity of the results: the large sample size reinforces the dependability of the research outcomes. Employing weighting mitigates biases stemming from oversampling. The consistent results across the main and sensitivity analyses suggest robustness in the findings. Analysing distinct subgroups enhanced data utilization and augmented the reliability of the conclusions.

### Limitations

The cross-sectional nature of the study underscores correlations but does not establish causality; thus, prospective research is imperative for validating causative relationships. The potential influence of unaccounted confounding factors cannot be entirely negated. While VCTE offers insights into liver steatosis and hepatic fibrosis, it is not the gold standard. A liver biopsy remains indispensable for a definitive diagnosis.

## Conclusion

The research demonstrates a positive correlation between the CMI and both NAFLD and liver fibrosis in the U.S. population. Given that CMI is a reproducible and easily measurable indicator, it holds considerable value in screening for NAFLD and fibrosis in adults.

## Data Availability

Publicly available datasets were analyzed in this study. This data can be found here: https://www.cdc.gov/nchs/nhanes.
